# 

**Published:** 1880-07-20

**Authors:** 


### TABLE OF CONTENTS.

ORIGINAL AND SELECTED ARTICLES.

Rhus Aromatica in Enuresis, by J. L. Lallerstedt, M.D., of Georgia, 233. Martin's Bandage, by W. W. Dawson, M.D., Cincinnati, 234. Intra-Uterine Medication, 237. Aural Syringes. by J. S. Prout, M.D , 239. Tuberculosis, an Infectious Disease, 242. Iodine a Substitute for Quinia, by Fordyce Grinnell, M.D.. Tenn., 245. Should we Bandage after Labor? by A. Elwell, M.D., N. J., 247. A [illegible]lea for Chloroform, 248.

ABSTRACTS AND GLEANINGS.

Local Anæsthesia with Bromide of Ethyl, 249. Sulphur in Skin Diseases, 250. Jamaica Dogwood; Oxalate of Cerium in Coughs, 251. Opium Habit—Possible Antidote, 252. Arsenic in Chorea, 253. Galvanic Current in Sciatica, 254. Hypodermic Injection of Ether in Sciatica; Nausea of Pregnancy—Flatulent Dyspepsia, 255. Removal of the Uterus, 256. Introduction of Liquids into Eustachian Tube and Middle Ear; Phosphate of Bismuth, 257. Successful Treatment of Imperforate Anus; Catarrh of the Bladder in Old People, 258. Carbolic Acid; Wickersheimer's Preservative Fluid, 259. Chloral Hydrate in Acute Gastro-Enteritis of Children; Ether and Chloroform, 260. Action of various Diuretics; Removing Particles of Steel, etc., from the Eye; New Operation for Prolapsus Uteri, 261. Oophorectomy; Prolapsus of the Rectum; Iodide of Potassium in Cough; Removing Nitrate of Silver Stains; Hemorrhoids Treated with Capsicum, 262.

SCIENTIFIC ITEMS.

Wormlike Derivatives from Blood Corpuscles; Dynamite for Stumps, 263. Light and Heat; Seeing by Telegraph, 264.

PRACTICAL NOTES AND FORMULÆ.

Salicylate of Potash; Remedies for Asthma; Tonic Wine, 265. The Diagnosis of Rotheln; Nervous Dyspepsia; Ergot in Pharyngitis, 266. Thymic Acid Mixture for Diphtheria; Formula for Gonorrhœa; Anodyne Mixture, 267. Dyspeptic Constipation; Treatment of Poisoning by Rhus Toxicodendron; Lactopeptine in Cholera Infantum, 268.

EDITORIAL AND MISCELLANEOUS.

Editorial Notices; Resignations and Changes; Our Journal, 269. Book Notices, 270. Special Notices, 272.

